# Elective neck dissection versus elective neck irradiation in cT3/4N0 maxillary sinus squamous cell carcinoma: a propensity score matching analysis

**DOI:** 10.1186/s12957-024-03368-8

**Published:** 2024-04-15

**Authors:** Min Chen, Hefeng Gu, Guihong Xuan, Lan Ma, Sunyu Tu, Min Li

**Affiliations:** Department of Stomatology, Shaoxing People’s Hospital, Zhejiang, PR China

**Keywords:** Maxillary sinus squamous cell carcinoma, Elective neck dissection, Elective neck irradiation, Quality of life, Prognosis

## Abstract

**Background:**

Maxillary sinus squamous cell carcinoma (MS-SCC) is an infrequent malignancy, and determining the optimal neck management for patients with cT3/4N0 MS-SCC remains a topic of ongoing debate. The purpose of this study was to compare the prognoses and quality of life outcomes of patients who underwent either elective neck dissection (END) or elective neck irradiation (ENI) for cT3/4N0 MS-SCC.

**Methods:**

In this retrospective study, we enrolled patients with surgically treated cT3/4N0 MS-SCC, and the impact of different neck management strategies on regional control and disease-specific survival was compared using propensity score matching. The effect of surgical intervention on quality of life was evaluated using the Mann-Whitney U test.

**Results:**

Of the 120 patients included, 36 underwent END. After propensity score matching, our analysis indicated that END did not lead to superior outcomes than ENI, as demonstrated by comparable rates of regional control (*p* = 0.990) and disease-specific survival (*p* = 0.999). However, in the 70 returned questionnaires, patients who underwent END reported higher scores in the domains of appearance, chewing, and speech than did patients who underwent ENI.

**Conclusions:**

Our findings suggest that while END and ENI contribute to similar prognoses, END yields superior functional outcomes.

## Introduction

Maxillary sinus squamous cell carcinoma (MS-SCC) is a rare malignancy, but it is the most common tumor of the paranasal sinuses. Due to early non-specific symptoms, it is often diagnosed at an advanced stage [1]. The curative treatment options for MS-SCC include primary site resection, with or without neck treatment, but optimal neck management for cT3/4N0 MS-SCC remains controversial [2].

While the NCCN guidelines indicate observation as a viable option, studies have shown the development of occult metastasis in a considerable percentage of cases [3], indicating that active intervention is necessary. Numerous studies have shown that elective neck treatment yields better cancer control and longer survival than observation alone [4–10]. However, it remains unclear whether there is a difference in prognosis between elective neck dissection (END) or elective neck irradiation (ENI). Furthermore, potential adverse impacts on quality of life (QoL) resulting from neck management - such as shoulder dysfunction following END or xerostomia after ENI - need to be considered [11].

Therefore, our objective is to compare the prognosis and QoL of patients who undergo END or ENI for cT3/4N0 MS-SCC.

## Methods

### Ethical approval

This study was approved by Shaoxing People Hospital Institutional Research Committee, and written informed consent for medical research was obtained from all patients before starting the treatment. All methods were performed in accordance with the relevant guidelines and regulations.

### Study design

Between January 2010 and December 2022, retrospective review of medical records was conducted for patients with primary MS-SCC treated by extended radical maxillectomy. The inclusion criteria were as follows: confirmation of stage cT3/4N0 using the 8th AJCC system, having undergone END or ENI, and availability of follow-up data for at least two years. Patients with a prior history of cancer were excluded. Data pertaining to demographics, pathology, treatment, and follow-up were collected and analyzed.

The Chinese version of the University of Washington Quality of Life (UW-QoL) questionnaire [12] was administered to each patient via email, post, clinic, telephone, or other forms of communication between January 2017 and December 2022.

### UW-QoL questionnaire

The UW-QoL scale consists of 12 single-question domains, each with 3 to 6 response options, evenly scaled from 100 (best) to 0 (worst) according to the hierarchy of response. The domains assessed were pain, appearance, activity, recreation, swallowing, chewing, speech, shoulder function, taste, saliva production, mood, and anxiety. A higher score on this scale indicates a better quality of life for the patient.

### Variable definition

The determination of cT3/4 was based on CT or MRI scans, while cN0 referred to the absence of clinically positive lymph nodes (LNs) as evaluated by ultrasound, CT, or MRI or PET/CT. The differentiation grade was categorized as well, intermediate, or poor. The presence of cancer cells within the lymphatic system was classified as lymphovascular invasion (LVI), while positive cancer cells within a nerve were defined as perineural invasion (PNI). The presence of cancer cells outside the LN capsule was considered extranodal extension (ENE).

Primary outcome variables were regional control (RC) and disease specific survival (DSS), time of RC was calculated from the date of surgery to the date of first regional recurrence or last follow-up, time of DSS was calculated from the date of surgery to the date of cancer caused death or last follow-up. The secondary outcome variable was the score obtained for each domain in the UW-QoL questionnaire.

### Treatment principle

In our cancer center, the treatment approach for individuals with cT3/4N0 MS-SCC typically involves a combination of surgery and radiation administered either before, after, or both before and after the primary surgical intervention. Primary tumor excision was attempted with a minimum 1 cm margin and reconstruction with flap was performed as needed, margin of soft tissue was determined using frozen section analysis, while the margin of bone was evaluated based on intraoperative findings. Adjuvant chemotherapy was suggested if there was presence of positive margin or ENE. A cN0 neck was treated with either END or ENI. The neck dissection procedure involved a minimum of level I-III/IV, with adjuvant radiotherapy performed when there was pathologic metastasis detected. Radiation fields for ENI consisted of at least ipsilateral level I-III/IV, while the contralateral neck was excluded.

### Statistic analysis

Clinicopathological variables were assessed utilizing the Chi-square test between END and ENI groups. The primary outcome variable evaluated the impact of different neck management approaches on RC and DSS using both univariate and Cox model analyses. The independent variables were further calculated using propensity score matching (PSM) at a 1:1 ratio between the END and ENI groups. Outcomes were presented in the form of a hazard ratio (HR) with a 95% confidence interval (CI). Comparison of quality of life (QoL) between the END and ENI groups - the secondary outcome variable - was conducted via the Mann-Whitney U test. All statistical analyses were carried out using R 3.4.3. A *p*-value below 0.05 was considered statistically significant.

## Results

### Baseline data

Altogether, 120 individuals with an average age of 50 ± 15 years were encompassed in the study, of whom 87 were male and 33 were female. PNI and LVI were observed in 43 and 33 subjects, respectively. Moreover, 16 individuals were classified as having well-differentiated tumors, 69 as having intermediate differentiation, and 35 as having poor differentiation. Notably, 47 cases were found to have positive margins following maxillectomy. The END and ENI groups exhibited comparable distribution of these clinicopathological variables (Table 1).

**Table 1:** Comparison of clinicopathologic variables between elective neck dissection (END) and elective neck irradiation (ENI) groups

Variable	END (*n* = 36)	ENI (*n* = 84)	*p*
Age			
<50	17	48	
≥50	19	36	0.318
Sex			
Male	27	60	
Female	9	24	0.688
PNI*			
Yes	13	30	
No	23	54	0.967
LVI^			
Yes	12	21	
No	24	63	0.349
Differentiation			
Well	5	11	
Intermediate	20	49	
Poor	11	24	0.960
Margin			
Positive	15	32	
Negative	21	52	0.713

*PNI: perineural invasion; ^LVI: lymphovascular invasion;

In the END group, fibula flap or anterolateral flap was utilized for maxillary reconstruction in 15 patients. Out of these, occult metastasis was found in 4 patients, with all positive LNs localized in level IIa and no evidence of ENE. Additionally, these 4 patients demonstrated poor differentiation and LVI. On the other hand, in the ENI group, all maxillary resections were performed without free flap reconstruction.

Over a median follow-up period of 3.2 years (range: 0.2–7.7 years), a total of 70 recurrences were identified, consisting of 63 local recurrences, 5 locoregional recurrences, and 2 regional recurrences. Additionally, there were 65 cancer-related deaths.

### Survival analysis

Univariate analysis revealed that differentiation and margin status were correlated with both RC and DSS, while PNI and LVI were found to be associated with DSS. These significant factors were subsequently incorporated into the Cox model. However, both END and ENI procedures exhibited a similar impact on RC and DSS, and neither age nor adjuvant chemotherapy were found to influence RC or DSS (Table 2).

**Table 2:** Univariate analysis of predictors for regional control (RC) and disease specific survival (DSS)

Variable	RC	DSS
Age (≥ 50 vs. < 50)	0.428	0.276
Sex (Male vs. Female)	0.153	0.398
PNI* (Yes vs. No)	0.187	0.006
LVI^ (Yes vs. No)	0.215	0.003
Differentiation (Poor vs. intermediate vs. well)	0.011	0.002
Margin (Positive vs. negative)	< 0.001	< 0.001
Neck management (END vs. ENI)^#^	0.135	0.432
Adjuvant chemotherapy (Yes vs. No)	0.638	0.354

*PNI: perineural invasion; ^LVI: lymphovascular invasion; # END: elective neck dissection; ENI: elective neck irradiation

In the multivariate analysis, a positive margin emerged as the most significant prognostic factor, with a HR of 3.22 [1.87–8.64] for RC and 9.03 [3.22–30.76] for DSS, as compared to well differentiation, intermediate differentiation carried comparable HRs for RC (1.75 [0.54–8.11]) and DSS (1.84 [0.65–4.78]), while poor differentiation was linked with a two-fold increased risk of recurrence (2.09 [1.43–7.29]) and a three-fold elevated risk of cancer-related mortality (3.26 [1.71–8.96]). Furthermore, the presence of LVI was associated with an additional one-fold possibility of death (1.92 [1.31–5.42]). (Table 3)

**Table 3:** Multivariate analysis of predictors for regional control (RC) and disease specific survival (DSS)

Variable	RC		DSS	
	*p*	HR [95%CI]	*p*	HR [95%CI]
PNI*				
No				Ref
Yes			0.108	2.20 [0.78–7.19]
LVI^				
No				Ref
Yes			0.023	1.92 [1.31–5.42]
Differentiation				
Well		Ref		Ref
Intermediate	0.367	1.75 [0.54–8.11]	0.286	1.84 [0.65–4.78]
Poor	0.014	2.09 [1.43–7.29]	0.002	3.26 [1.71–8.96]
Margin				
Negative		Ref		Ref
Positive	0.008	3.22 [1.87–8.64]	< 0.001	9.03 [3.22–30.76]

*PNI: perineural invasion; ^LVI: lymphovascular invasion; # END: elective neck dissection; ENI: elective neck irradiation

### PSM analysis

**Fig. 1:**
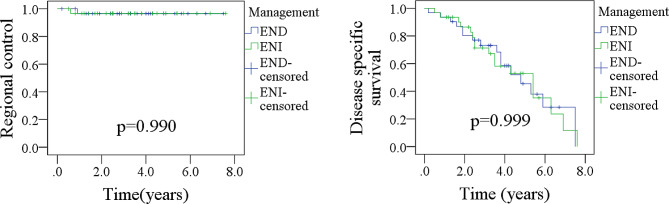
Comparison of regional control and disease specific survival in patients treated by elective neck dissection (END) and elective neck irradiation (ENI) after propensity score matching

To mitigate the potential impact of confounding variables, PSM analysis was conducted using variables of LVI, differentiation, and margin status. In total, 66 patients (33 in each group) were included. The results indicated that END did not yield superior outcomes compared to ENI, as evidenced by comparable rates of RC (*p* = 0.990) and DSS (*p* = 0.999). (Fig. 1; Table 4).

**Table 4:** Univariate analysis of predictors for regional control (RC) and disease specific survival (DSS) after prosperity score matching

Variable	RC	DSS
Age (≥ 50 vs. < 50)	0.356	0.189
Sex (Male vs. Female)	0.876	0.478
PNI* (Yes vs. No)	0.327	0.018
LVI^ (Yes vs. No)	0.113	0.011
Differentiation (Poor vs. intermediate vs. well)	0.037	0.017
Margin (Positive vs. negative)	0.043	0.035
Neck management (END vs. ENI)^#^	0.990	0.999
Adjuvant chemotherapy (Yes vs. No)	0.328	0.548

*PNI: perineural invasion; ^LVI: lymphovascular invasion; # END: elective neck dissection; ENI: elective neck irradiation

### QoL

Out of a total of 120 questionnaires distributed, 70 were returned, giving a response rate of 58.3%. Among these, 20 questionnaires were completed by participants in the END group, while the remaining 50 were completed by individuals in the ENI cohort. The END group achieved significantly higher scores in appearance (60 ± 21), chewing (74 ± 14), and speech (70 ± 15), as compared to the ENI group (*p* = 0.016, *p* = 0.007, and *p* = 0.011, respectively). The two groups showed comparable scores in other domains (all *p* > 0.05). (Table 5)

**Table 5:** Quality of life in elective neck dissection (END) and elective neck irradiation (ENI) groups

Domain	END (*n* = 20)	ENI (*n* = 50)	*p*
Pain	50 ± 18	47 ± 20	0.685
Appearance	60 ± 21	41 ± 15	0.016
Activity	63 ± 13	58 ± 19	0.796
Recreation	74 ± 25	72 ± 16	0.888
Swallowing	64 ± 20	58 ± 18	0.659
Chewing	74 ± 14	51 ± 12	0.007
Speech	70 ± 15	48 ± 17	0.011
Shoulder	78 ± 21	85 ± 18	0.587
Taste	58 ± 22	64 ± 23	0.499
Saliva	42 ± 15	35 ± 13	0.721
Mood	55 ± 26	61 ± 18	0.643
Anxiety	54 ± 23	62 ± 19	0.668

## Discussion

Undoubtedly, our most significant discovery was that cT3/4N0 MS-SCC typically portended a poor prognosis, with death often being the result of local invasion, while regional recurrence was relatively infrequent. Although END did not prove advantageous in terms of RC or DSS when compared to ENI, it did offer superior outcomes with respect to appearance, chewing, and speech. Our findings have practical implications for the development of a more effective treatment plan for cT3/4N0 MS-SCC, aiding clinicians in providing optimal care for their patients.

The challenge of treating cT3/4N0 MS-SCC is further compounded by its low incidence, as well as ongoing debates regarding optimal neck management despite widespread agreement on primary tumor resection. Historically, due to the assumed low incidence of occult metastasis, emphasis was placed on local control, though recent evidence reveals that LN metastasis is more common than previously believed [4], with untreated necks experiencing regional failure in up to 33% of cases [13]. Through an analysis of oncologic outcomes among 777 N0 MS-SCC patients, Li et al. [7] demonstrated that those who underwent END displayed significantly improved overall survival and DSS compared to their counterparts who did not undergo END. Furthermore, when combined with radiotherapy, END was even more effective in promoting a favorable prognosis. Abu-Ghanem et al. [4], in a review of four retrospective studies comprising a total of 129 patients, determined that ENI significantly reduced the risk of regional nodal recurrence by nearly 80% compared to observation. Similarly, Faisal et al. [14], in an RC assessment of 255 patients with MS-SCC, found that positive LNs were present in 14.1% of the population, with regional relapse occurring in 3.7% of those who underwent elective neck treatment, compared to 26.4% in those who did not. Elective neck treatment, therefore, considerably reduced the risk of regional recurrence. In a multicenter study conducted in Japan [3], 111 patients were treated with curative intent, out of which 98 had cN0 neck disease and did not receive prophylactic neck irradiation. Subsequently, 11 patients (11.2%) were diagnosed with lymph node metastasis, of which eight formed part of the 83 patients with an N0 neck who had not undergone elective neck treatment. Taken together, these findings reveal that END and ENI deliver better prognoses compared to observation, however, the question as to whether END is superior to ENI remains unanswered until our current investigation, which shows no significant differences in terms of RC and DSS between END and ENI. The lower occurrence rate of occult metastasis in our study (11.1%) compared to earlier studies [4–10] may be attributed to the utilization of PET/CT to confirm cN0 status in most of our patients, thereby enabling prompt intervention to easily control the very few positive LNs through subsequent END or ENI [1].

Evaluating the impact of a therapeutic procedure on QoL is another crucial aspect to consider. While END may be technically mature and associated with minimal complications when carried out by an experienced practitioner, some patients have reported complaints of shoulder dysfunction [15]. Although radiation therapy techniques are constantly improving, it is important not to disregard potential complications such as dry mouth [16]. Our study may represent the first comparison of QoL in patients who underwent END and ENI, and we observed that those who received END reported higher scores with regards to appearance, chewing, and speech. Our findings are particularly intriguing, as they shed light on the critical role that the maxilla plays in maintaining both oral function and facial appearance. Notably, cT3/4 MS-SCC often necessitates extensive resection of the maxilla, which can result in facial collapse and the development of a communication between the oral and nasal cavities. Furthermore, it is worth noting that a small number of patients in the END group received neck radiotherapy, which could have had a significant impact on the differences in QoL observed between the two groups.

However, it is important to carefully consider the advantages and disadvantages of elective neck treatment. First, there is general consensus that surgery and/or radiotherapy on the neck is indicated for patients with clinically positive nodes. However, in patients with a tumor that has a low rate of LN metastases at presentation, preventive treatment of the neck may not be necessary. In fact, Cantù et al. [17] demonstrated that subsequent LN metastases in cN0 patients were rare and could be effectively treated with careful monitoring and follow-up. In their study, only 2 out of 182 patients with MS-SCC died from nodal metastases. Similarly, in the study by Patel et al., 15.4% of patients did not receive any treatment of the neck, 2.6% underwent an elective neck dissection (END), 69.2% received elective nodal irradiation (ENI) only, and 12.8% were treated with both END followed by radiotherapy. Notably, none of the patients with N0 necks had isolated regional recurrence regardless of neck management [1]. Second, it is important to remember that approximately 80–90% of cN0 patients may undergo unnecessary preventive neck treatment, despite potential complications associated with such treatment. Third, even with spinal accessory nerve-preserving neck dissections, patients may experience variable degrees of shoulder dysfunction and must be informed of the associated risks before undergoing treatment. Furthermore, while radiation therapy techniques are improving, potential complications such as dry mouth must not be ignored [18, 19]. Thus, it is imperative to identify the factors that can inform decision-making regarding elective neck treatment. Existing evidence suggests that lymph node (LN) metastases are more frequently observed in tumors involving the palate and upper gum, and that these tumors behave more similarly to tumors of the oral cavity than those of the maxilla [17, 20]. It is therefore essential to consider the site and characteristics of the primary tumor when determining the need for and extent of elective neck treatment, however, due to the retrospective nature of our study and its extended duration, accurately assessing invasion of the palate and upper gum may be challenging.

Poor differentiation and positive margin were the two most important prognostic factors. Wang et al. [21] also described that low differentiation was related to both additional increased 50% risk of overall death and cancer-caused death. Positive margin was common during surgical treatment of advanced stage MS-SCC, and it certainly predicted worse survival [22].

Limitation in current study must be acknowledged, first, there was inherent bias within retrospective study, second, the response rate of QoL questionnaire was not very satisfactory, it might decrease our statistic power, third, more external validation was required before clinical application.

## Conclusion

In summary, patients with cT3/4N0 MS-SCC typically face a poor prognosis, and while both END and ENI contribute to similar prognoses, but END offers better functional outcomes.

## Data Availability

All data generated or analyzed during this study are included in this published article. And the primary data could be achieved from the corresponding author.

## References

[1] Patel EJ, Strohl MP, Yom SS, El-Sayed I (2024). Elective management of the N0 neck in maxillary sinus squamous cell carcinoma. Head Neck.

[2] Byrd JK, Clair JM, El-Sayed IAHNS, Series (2018). Do you know your guidelines? Principles for treatment of cancer of the paranasal sinuses: a review of the National Comprehensive Cancer Network guidelines. Head Neck.

[3] Homma A, Hayashi R, Matsuura K, Kato K, Kawabata K, Monden N, Hasegawa Y, Onitsuka T, Fujimoto Y, Iwae S, Okami K, Matsuzuka T, Yoshino K, Nibu K, Kato T, Nishino H, Asakage T, Ota I, Kitamura M, Kubota A, Ueda T, Ikebuchi K, Watanabe A, Fujii M (2014). Lymph node metastasis in t4 maxillary sinus squamous cell carcinoma: incidence and treatment outcome. Ann Surg Oncol.

[4] Abu-Ghanem S, Horowitz G, Abergel A, Yehuda M, Gutfeld O, Carmel NN, Fliss DM (2015). Elective neck irradiation versus observation in squamous cell carcinoma of the maxillary sinus with N0 neck: a meta-analysis and review of the literature. Head Neck.

[5] Peng H, Ye MC, Wang LP, Li RX, Zhou Y, Wang Y, Zhu WZ (2015). [Analysis of the outcomes of squamous cell carcinoma of maxillary sinus with 3 different comprehensive treatments]. Shanghai Kou Qiang Yi Xue.

[6] Sangal NR, Lee YJ, Brady JS, Patel TD, Eloy JA, Baredes S, Park RCW (2018). The role of elective neck dissection in the treatment of maxillary sinus squamous cell carcinoma. Laryngoscope.

[7] Li H, Song Y, Zhao L, Liu Y, Liu S (2023). Role of elective neck dissection in prognosis of N0M0 maxillary sinus squamous cell carcinoma: insights from SEER database analysis. J Stomatol Oral Maxillofac Surg.

[8] Berger MH, Tajudeen BA, St John MA, Tjoa T, Kuan EC (2019). Should an elective neck dissection be performed for maxillary sinus squamous cell carcinoma?. Laryngoscope.

[9] Jeon SH, Han DH, Won TB, Keam B, Kim JH, Wu HG (2017). Implication of Tumor Location for Lymph Node Metastasis in Maxillary Sinus Carcinoma: indications for Elective Neck Treatment. J Oral Maxillofac Surg.

[10] Thawani R, Kim MS, Arastu A, Feng Z, West MT, Taflin NF, Thein KZ, Li R, Geltzeiler M, Lee N, Fuller CD, Grandis JR, Floudas CS, Heinrich MC, Hanna E, Chandra RA (2023). The contemporary management of cancers of the sinonasal tract in adults. CA Cancer J Clin.

[11] Farlow JL, McLean SA, Peddireddy N, Bradford CR, Malloy KM, Stucken CL, VanKoevering KK, Spector ME, Rosko AJ (2022). Impact of Completion Lymphadenectomy on Quality of Life for Head and Neck cutaneous melanoma. Otolaryngol Head Neck Surg.

[12] Fang QG, Shi S, Zhang X, Li ZN, Liu FY, Sun CF. Assessment of the quality of life of patients with oral cancer after pectoralis major myocutaneous flap reconstruction with a focus on speech. J Oral Maxillofac Surg. 2013; 71:2004.e1-2004.e5. 10.1016/j.joms.2013.07.011 24135522

[13] Takes RP, Ferlito A, Silver CE, Rinaldo A, Medina JE, Robbins KT, Rodrigo JP, Hamoir M, Suárez C, Zbären P, Mondin V, Shaha AR, Mendenhall WM, Strojan P (2014). The controversy in the management of the N0 neck for squamous cell carcinoma of the maxillary sinus. Eur Arch Otorhinolaryngol.

[14] Faisal M, Seemann R, Lill C, Hamzavi S, Wutzl A, Erovic BM, Janik S (2020). Elective neck treatment in sinonasal undifferentiated carcinoma: systematic review and meta-analysis. Head Neck.

[15] McDonald C, Kent S, Schache A, Rogers S, Shaw R (2023). Health-related quality of life, functional outcomes, and complications after sentinel lymph node biopsy and elective neck dissection in early oral cancer: a systematic review. Head Neck.

[16] Lindegaard AM, Håkansson K, Bernsdorf M, Gothelf AB, Kristensen CA, Specht L, Vogelius IR, Friborg J (2023). A systematic review on clinical adaptive radiotherapy for head and neck cancer. Acta Oncol.

[17] Cantù G, Bimbi G, Miceli R, Mariani L, Colombo S, Riccio S, Squadrelli M, Battisti A, Pompilio M, Rossi M (2008). Lymph node metastases in malignant tumors of the paranasal sinuses: prognostic value and treatment. Arch Otolaryngol Head Neck Surg.

[18] Gane EM, Michaleff ZA, Cottrell MA, McPhail SM, Hatton AL, Panizza BJ, O’Leary SP (2017). Prevalence, incidence, and risk factors for shoulder and neck dysfunction after neck dissection: a systematic review. Eur J Surg Oncol.

[19] Shah K, Patekar S, Ishwarya M, Padmakshan S, Bradoo R (2023). Shoulder dysfunction post spinal accessory nerve preserving Neck dissections: our experience. Indian J Otolaryngol Head Neck Surg.

[20] Givi B, Eskander A, Awad MI, Kong Q, Montero PH, Palmer FL, Xu W, De Almeida JR, Lee N, O’Sullivan B, Irish JC, Gilbert R, Ganly I, Patel SG, Goldstein DP, Morris LG (2016). Impact of elective neck dissection on the outcome of oral squamous cell carcinomas arising in the maxillary alveolus and hard palate. Head Neck.

[21] Wang Y, Yang R, Zhao M, Guo W, Zhang L, Zhang W, Wang X (2020). Retrospective analysis of 98 cases of maxillary sinus squamous cell carcinoma and therapeutic exploration. World J Surg Oncol.

[22] Bahig H, Ehab HY, Garden AS, Ng SP, Frank SJ, Nguyen T, Gunn GB, Rosenthal DI, Fuller CD, Ferrarotto R, Bell D, Su S, Phan J (2023). Long-term outcomes of modern multidisciplinary management of sinonasal cancers: the M. D. Anderson experience. Head Neck.

